# Hierarchical two-stage ensemble of dilated U-net models for brain tumor segmentation

**DOI:** 10.1186/s42492-026-00230-4

**Published:** 2026-08-26

**Authors:** Vladyslav Koniukhov

**Affiliations:** Laboratory of Quality Management of Scientific Research and Scientific Marketing, National Scientific Center “Institute of Experimental and Clinical Veterinary Medicine”, Hryhoriia Skovorody, Kharkiv Region Kharkiv 61023, Ukraine

**Keywords:** Brain tumor segmentation, Deep learning, Medical image analysis, Image processing, Hierarchical ensemble learning

## Abstract

Ensemble methods for image segmentation improve performance by combining predictions from multiple models, yielding more accurate and reliable results. This study presents a two-stage hierarchical framework to enhance the accuracy and stability of brain tumor delineation in magnetic resonance imaging data. The proposed approach integrates ensemble strategies at different stages of the processing pipeline. The architecture operates in two stages: first, sub-ensembles resolve internal inconsistencies through simple averaging; second, their outputs are fused into a final prediction using union-based aggregation. The method was evaluated on the Figshare brain tumor dataset and demonstrated progressive performance improvements from individual models to the final hierarchical ensemble. The proposed approach achieved a Dice coefficient of 94.50% and an intersection over union of 89.91%, outperforming existing state-of-the-art methods. The statistical significance of these improvements was confirmed using one-way analysis of variance across three experimental groups, followed by post hoc pairwise testing. The proposed architecture preserves high fidelity in delineating diffuse tumor boundaries and complex morphological structures. By decomposing the ensemble process into two stages, the framework effectively reduces stochastic errors typical of single-model predictions, resulting in a more robust and stable segmentation system that performs reliably even in cases with low contrast and complex tissue interfaces.

## Introduction

The brain is a vital organ responsible for the functioning of the central nervous system. Brain health is a crucial component of overall health, making its assessment and monitoring an important part of modern healthcare systems in developed countries. Protecting and promoting brain health require a holistic approach throughout the lifespan [1]. This perspective is consistent with the World Health Organization’s position that optimizing brain health can reduce the prevalence of neurological disorders while also improving mental and physical health [2].

Although adherence to recommendations for maintaining brain health contributes significantly to overall well-being, various diseases can substantially impair brain function. According to recent research, the ten major brain diseases are stroke, neonatal encephalopathy, migraine, dementia, diabetic neuropathy, meningitis, idiopathic epilepsy, neurological complications of preterm birth, autism spectrum disorder, and nervous system cancer [3]. Among these diseases, brain tumors are of particular clinical significance because, although less common, they have a high clinical impact. Early diagnosis of brain tumors is a key focus in neuro-oncology. According to 2025 statistics, the estimated number of malignant tumors of the brain and other nervous system organs is 24,820 cases, with a projected 18,330 deaths. Based on mortality data from 2022, brain tumors are the leading cause of cancer-related deaths among children and adolescents under 20 years of age. Among men aged 20–39 years, brain tumors are also a leading cause of cancer-related mortality [4].

Magnetic resonance imaging (MRI) plays a key role in brain tumor analysis. It offers several advantages over its primary alternative, computed tomography. These advantages include excellent soft tissue contrast, high spatial resolution, the absence of ionizing radiation, multiplanar imaging capability, high sensitivity to small tumors, and fewer imaging artifacts [5]. These characteristics establish MRI as an indispensable imaging modality for brain tumor diagnosis. However, manual analysis of MRI scans is labor-intensive, time-consuming, and dependent on subjective human interpretation. Consequently, manual assessment is prone to errors, interobserver variability, and the omission of subtle pathological changes, limiting its reliability for clinical use. To improve segmentation quality and reduce physicians’ analysis time, various classical methods have been proposed, including Otsu’s method, watershed, level set, K-means, and discrete wavelet transform using Haar wavelets. Despite the numerous advantages of MRI, brain tumor segmentation using classical computer vision methods remains a labor-intensive and challenging task. A comparative study of classical methods and neural network-based approaches highlighted the substantial advantages of the latter [6].

Deep learning models have consistently demonstrated superior performance over traditional machine learning approaches [7]. Unlike conventional algorithms that require manual feature extraction, deep neural networks automatically learn relevant feature representations from MRI scans. However, deep learning methods require large annotated datasets and remain susceptible to model-specific errors. To address these limitations and improve segmentation accuracy, ensemble methods have been increasingly adopted. Their strong generalization capability and the complementary strengths of multiple models enable further improvements in segmentation accuracy [8].

This study proposes an approach based on the integration of multiple ensemble strategies. The proposed framework reduces the impact of individual model weaknesses as well as the inherent limitations of single ensemble strategies. The study demonstrates that the proposed multi-level ensemble framework not only improves overall segmentation accuracy but also minimizes the likelihood of clinically significant segmentation errors, such as large false-negative regions.

### Related work

Automating MRI scan analysis is motivated by the high labor costs of manual segmentation and the clinical need for noninvasive diagnostic methods. The literature identifies three main methodological generations: region growing, classical machine learning, and deep learning [9]. Over the past decade, the focus has gradually shifted from classical methods to deep learning and, more recently, to ensemble methods, which have demonstrated state-of-the-art segmentation accuracy. However, brain tumor segmentation remains challenging because of the substantial variability in tumor size, shape, and intensity patterns across patients.

Before the advent of deep learning, MRI segmentation was primarily performed using classical computer vision methods. One of the earliest approaches was thresholding, which was effective for homogeneous regions but highly sensitive to noise [6, 10–12]. The watershed method, often combined with thresholding, has also been widely applied to brain tumor segmentation [13–15].

Much of the research has focused on hybrid methods that combine classical segmentation techniques with clustering algorithms to improve accuracy. Abdel-Maksoud et al. [16] proposed a multi-stage approach combining K-means with fuzzy C-means. This approach provides higher segmentation accuracy by better handling fuzzy and ambiguous region boundaries. El-Melegy and Mokhtar [17] proposed a modified fuzzy clustering approach based on a priori information about class centers. Furthermore, Mayala et al. [18] proposed a semi-automatic method for brain tumor segmentation based on the minimum spanning tree.

A major breakthrough in medical image segmentation was the introduction of the U-Net architecture [19]. The framework introduced by its authors quickly became the de facto standard architecture for medical image segmentation and has served as the foundation for numerous subsequent studies. In recent years, deep learning has become the dominant approach in medical image analysis, largely replacing classical computer vision methods [20]. Alquran et al. [21] performed a comparative analysis of various U-Net architectural modifications. Architectures based on vision Transformers have demonstrated superior performance over conventional convolutional neural networks because of their ability to capture long-range dependencies [22]. Preetha et al. [23] proposed a multiscale attention U-net model integrated with the EfficientNetB4 encoder, enabling multi-scale feature extraction while maintaining low computational cost. Jin et al. [24] addressed the problem of limited annotated medical data. In their review, they systematized brain tumor segmentation methods that use both labeled and unlabeled MRI samples.

Incremental transfer learning methods enable models to improve performance under conditions of limited medical data [25]. Sobhaninia et al. [26] proposed a cascade approach using multi-scale images to simultaneously extract local and global contextual representations. Díaz-Pernas et al. [27] proposed a fully automatic multiscale deep convolutional neural network for brain tumor segmentation that processes MRI scans at three spatial scales, mimicking the human visual system.

The theoretical foundation for the superiority of ensemble models over individual models was established in a seminal study on ensemble methods [28]. Khan et al. [29] demonstrated the feasibility of complex ensemble systems for stable clinical segmentation. Dang et al. [30] proposed a two-layer deep learning ensemble in which the outputs of first-layer models serve as input features for training the second layer. A growing trend in brain tumor segmentation is the use of ensemble methods that combine predictions from multiple models to minimize errors. This strategy improves segmentation accuracy and robustness compared with any individual model [31–36].

## Methods

### Dataset

**Fig. 1:**
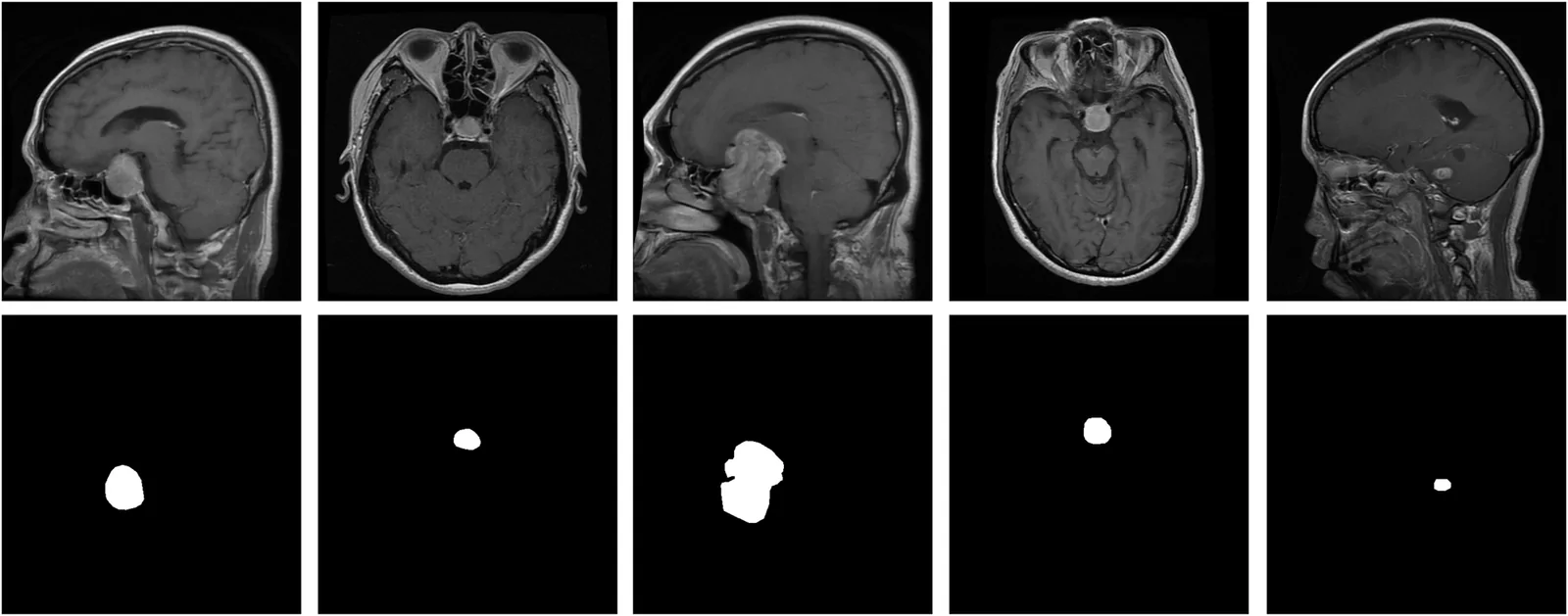
Sample magnetic resonance imaging scans and corresponding segmentation masks

This study used an expanded open brain MRI dataset [37], comprising 3064 T1-weighted contrast-enhanced MRI images from 233 patients. The dataset includes three representative tumor types: glioma (1426 slices), pituitary adenoma (930 slices), and meningioma (708 slices). A visual representation of the raw images and the corresponding expert-annotated masks is shown in Fig. 1.

All images were acquired between September 2005 and October 2010 at Nanfang Hospital (Guangzhou) and the General Hospital of Tianjin Medical University (China). The imaging protocol included the administration of a gadolinium-based contrast agent at a dose of 0.1 mmol/kg and an injection rate of 2 mL/s.

The source data were distributed across four archives (766 slices each) and stored in MATLAB (.mat) format. For compatibility with the deep learning pipeline, the images were converted to PNG format while preserving the original resolution of *512 × 512* pixels.

For model training, all structural images underwent pixel-intensity normalization, with pixel values rescaled from the original [0, 255] range to [0, 1] using min–max rescaling. No data augmentation was applied during training. This constraint was intentionally introduced to evaluate the intrinsic architectural capability of the hierarchical ensemble to suppress noise without synthetic data inflation.

The 80/20 dataset split configuration was selected to balance model optimization requirements with the reliability of statistical evaluation. The training subset (80%, 2451 images) provides sufficient sample diversity for the 20 baseline models to achieve stable convergence. Reserving 10% of the training samples for validation enables unbiased early stopping to prevent overfitting. The remaining 20% of the data (613 images) constitutes a completely independent test set. This set was excluded from all weight optimization procedures and was used exclusively for the final evaluation of segmentation performance.

### Implementation details and hardware setup

All experiments were conducted in a 64-bit Windows 11 Pro environment on a local workstation. The hardware configuration consisted of an Intel Core i7-10700 CPU (2.90 GHz), 32 GB of DDR4 RAM, and an NVIDIA GeForce RTX 4060 Ti GPU equipped with 16 GB of dedicated VRAM. The software pipeline was developed in Python using the TensorFlow GPU (v2.9.1) and Keras (v2.9.0) frameworks. GPU acceleration was enabled using NVIDIA CUDA 11.2 and cuDNN 8.1.1.

### Model configuration and hyperparameters

For tumor segmentation, the Dilated U-net architecture [38] was trained using the Adam optimizer with a binary cross-entropy loss function. The Dice coefficient and intersection over union (IoU) were monitored as the primary metrics for evaluating model performance during training.

The neural network was trained for 100 epochs with a batch size of 2. To improve the model’s generalization capability and prevent overfitting, an early stopping strategy was implemented. The Dice coefficient on the validation set (10% of the training set) was monitored with the objective of maximizing this metric. The patience parameter was set to 10 epochs, after which training was automatically terminated if the target metric failed to improve. Upon completion of training, the model weights corresponding to the best validation performance were restored. The hyperparameters used for model training are presented in Table  1.

**Table 1:** Hyperparameters used for model training

Hyperparameter	Value
Input image size	*512 × 512*
Batch size	2
Number of epochs	100
Learning rate	$1 \times 10^{-3}$
Optimizer	Adam
Loss function	Binary cross-entropy
Activation function	ReLU, Sigmoid
Early stopping patience	10

To evaluate the computational complexity of the proposed pipeline, structural metrics were extracted for the baseline Dilated U-net architecture. Each standalone model contains 31,054,145 parameters, including 31,042,369 trainable parameters and 11,776 non-trainable parameters. The resulting trained model occupies 118.62 MB of storage space.

### Post-processing

To improve segmentation accuracy and remove small isolated regions misclassified as pathology, a post-processing step was applied to the final output of the ensemble. Contour-based topological analysis was performed using OpenCV’s findContours function, which implements the border-following algorithm proposed by Suzuki and Abe [39]. Contours were extracted using the RETR_TREE retrieval mode and the CHAIN_APPROX_NONE approximation method. Based on the a priori knowledge that the target brain tumor in each MRI slice forms a single dominant connected structure, the filtering mechanism computes the area of all detected regions and retains only the component corresponding to the largest contour area, while automatically discarding all smaller disconnected artifacts.

### Proposed method

The study was conducted in three stages: evaluation of individual models, construction of baseline ensembles, and implementation of the proposed hierarchical workflow, followed by comprehensive ablation studies of both the ensemble configuration and the post-processing step.

In the first stage, a set of 20 individual models was trained independently using the same training dataset and preprocessing pipeline. The ensemble size of 20 models was selected based on the deep learning literature [40] and the author’s prior empirical findings [41]. Standard deep ensemble frameworks demonstrate that predictive accuracy and uncertainty calibration improve with a relatively small number of models (typically *M=5* to *M=10*), while performance gains gradually plateau beyond this range. This trend was verified in our previous work on X-ray image segmentation. Therefore, 20 models were selected as a practical compromise between prediction accuracy and computational cost. Although all models share the same architecture and training data, they diverge because of multiple independent sources of stochasticity inherent in the training process, including random weight initialization, mini-batch shuffling order, and variations in the composition of the validation subset used for early stopping.

In the second stage, ensemble learning was applied to improve overall predictive performance. Two baseline ensemble approaches were constructed using all 20 trained models.

In the third stage, a novel two-stage ensemble workflow was proposed, building upon the baseline ensembles by organizing them into a hierarchical structure.

#### Simple averaging method

In this approach, the final prediction is obtained by averaging the binary masks produced by all models in the ensemble. For an ensemble of *M* trained models, each model produces a binary segmentation mask. Let $B_k(i,j) \in \{0,255\}$ denote the predicted mask value of the *k*-th model for pixel *(i,j)*.

First, the masks are averaged across all models:

1 $$\bar{B}(i,j) = \frac{1}{M} \sum_{k=1}^{M} B_k(i,j)$$

Then, a thresholding operation is applied to obtain the final binary segmentation mask:

2 $$E(i,j)=\begin{cases}255, & \bar{B}(i,j) > T\\0, & \mathrm{otherwise}\end{cases}$$

where *M* is the number of models in the ensemble, *T* is a predefined threshold (typically *T = 128*), and *E(i,j)* is the final predicted mask value for pixel *(i,j)*.

#### Union-based aggregation method

The union-based aggregation method combines binary predictions from multiple models by assigning a pixel to the target class if at least one model predicts it as belonging to that class.

Let $B_k(i,j) \in \{0,255\}$ denote the binary prediction of the *k*-th model for pixel *(i,j)*, where 0 corresponds to the background and 255 to the target class.

The final prediction is obtained as:

3 $$E(i,j) = \max_{k \in \{1,\dots,M\}} B_k(i,j)$$

This operation is equivalent to a pixel-wise logical OR applied to the binary masks, where *M* is the number of models in the ensemble and *E(i,j)* is the final predicted mask value for pixel *(i,j)*.

#### Two-stage ensemble workflow

The proposed approach combines multiple ensemble models into a hierarchical two-stage workflow. Specifically, the 20 trained models were divided into four groups, each containing five models. These groups define the first-stage aggregation level. For each group, an intermediate ensemble was constructed using an aggregation method $A_1$. Let $G_j(x)$ denote the output of the *j*-th ensemble (where *j=1,2,3,4*):

4 $$G_j(x)=A_1\big(S_{j,1}(x),\ldots, S_{j,5}(x)\big)$$

where $S_{j,k}(x)$ denotes the predicted mask of the *k*-th model in the *j*-th group, and $A_1$ is the aggregation function applied within each group.

The second stage aggregates the outputs of the four first-stage ensembles using an aggregation method $A_2$:

5 $$S_{\mathrm{final}}(x)=A_2\big(G_1(x),G_2(x),G_3(x),G_4(x)\big)$$

This hierarchical aggregation defines the proposed method. Four variants of the proposed approach were investigated:

1. *A*_1_ = Simple averaging, *A*_2_ = Simple averaging
2. *A*_1_ = Union-based aggregation, *A*_2_ = Union-based aggregation
3. *A*_1_ = Union-based aggregation, *A*_2_ = Simple averaging
4. *A*_1_ = Simple averaging, *A*_2_ = Union-based aggregation

**Fig. 2:**
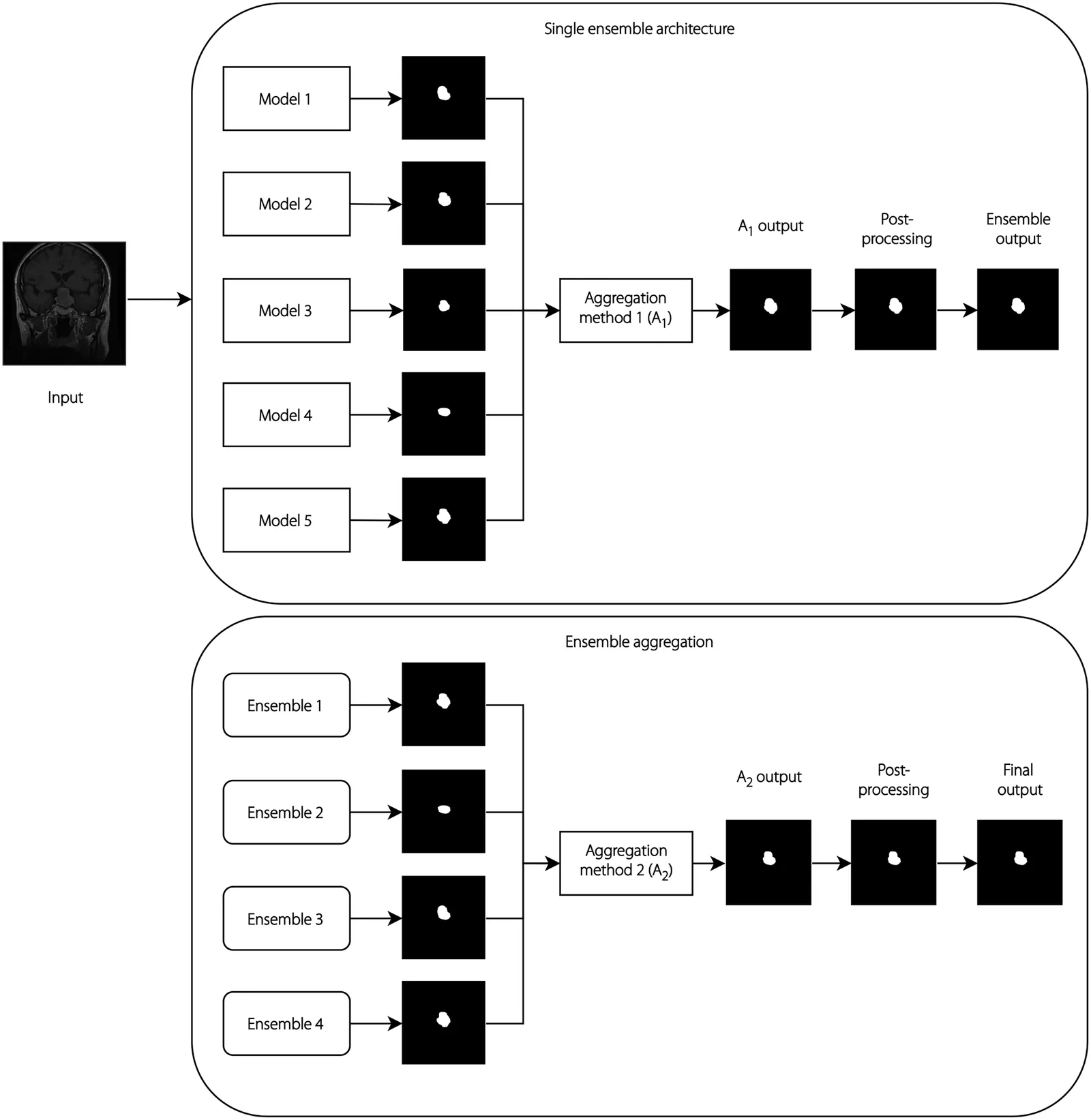
Proposed two-stage ensemble workflow

Each variant represents a different combination of aggregation strategies across the two stages of the ensemble. A schematic representation of the proposed two-stage ensemble workflow is shown in Fig. 2.

Finally, it should be noted that the post-processing procedure described in the previous subsection is applied to every predicted mask at all stages of the proposed workflow, including both intermediate ensemble outputs and the final prediction.

#### Ablation studies

An ablation study was conducted to evaluate the grouping strategy and verify the ensemble’s invariance to the model order. Twenty models were tested across five configurations: one group of 20, two groups of 10, four groups of five, five groups of four, and ten groups of two. Across all tested configurations, the intragroup predictions were combined using simple averaging, whereas the final intergroup consensus was achieved via union-based aggregation. For each configuration, the sequence of models was randomly permuted across independent trials. This setup ensured that the effectiveness of the threshold-averaging mechanism was measured independently of the models’ chronological training sequence. In addition, to quantify the structural contribution of the postprocessing stage, the performance of the pipeline was evaluated with and without the morphological filtering step.

### Performance evaluation metrics

To quantitatively evaluate the performance of the proposed method and the baseline models, several widely used image segmentation metrics were employed, including the Dice coefficient, IoU, accuracy, recall, precision, and specificity. Let true positives (TP), true negatives (TN), false positives (FP), and false negatives (FN) denote the number of correctly classified positive pixels, correctly classified negative pixels, incorrectly classified positive pixels, and incorrectly classified negative pixels, respectively. The Dice coefficient measures the overlap between the predicted and ground truth masks, and is defined as follows:

6 $$\mathrm{DSC}=\frac{2 TP}{2 TP+FP+FN}$$

The IoU metric measures the ratio of the intersection to the union of the predicted and ground truth regions:

7 $$\mathrm{IoU}=\frac{TP}{TP+FP+FN}$$

Accuracy represents the proportion of correctly classified pixels:

8 $$\mathrm{Accuracy}=\frac{TP+TN}{TP+TN+FP+FN}$$

Recall measures the ability of the model to correctly identify positive pixels:

9 $$\mathrm{Recall}=\frac{TP}{TP+FN}$$

Precision represents the proportion of correctly predicted positive pixels among all predicted positive pixels:

10 $$\mathrm{Precision}=\frac{TP}{TP+FP}$$

Specificity measures the ability of the model to correctly identify negative pixels:

11 $$\mathrm{Specificity}=\frac{TN}{TN+FP}$$

## Results and Discussion

### Ensemble performance and ablation

In the first stage of the study, 20 independent models based on the Dilated U-net architecture were evaluated, and the results are presented in Table  2. A comparative analysis based on descriptive statistics was performed to assess model performance. The average Dice coefficient across all models was 90.16%, whereas the average IoU was 84.28%. Overall accuracy was also notably high, with a mean value of 99.72%.

**Table 2:** Performance of individual models (*mean ± SD*, %)

Model	Dice	Intersection over union	Accuracy	Recall	Precision	Specificity
1	88.70 *±* 16.05	82.13 *±* 17.35	99.68 *±* 00.43	87.76 *±* 17.84	92.80 *±* 10.92	99.89 *±* 00.18
2	90.16 *±* 11.80	83.61 *±* 14.48	99.71 *±* 00.37	89.35 *±* 13.50	93.09 *±* 10.13	99.89 *±* 00.16
3	93.61 *±* 10.44	89.20 *±* 12.60	99.80 *±* 00.42	**93.49 ***±*** 12.43**	95.09 *±* 06.62	99.93 *±* 00.12
4	93.23 *±* 10.20	88.47 *±* 12.25	99.78 *±* 00.39	93.23 *±* 11.43	94.13 *±* 09.73	99.91 *±* 00.16
5	**93.61 ***±*** 10.43**	**89.24 ***±*** 13.02**	**99.81 ***±*** 00.30**	93.37 *±* 11.76	95.02 *±* 08.83	99.93 *±* 00.13
6	91.47 *±* 12.07	85.83 *±* 14.35	99.75 *±* 00.34	90.57 *±* 13.65	94.22 *±* 09.71	99.91 *±* 00.16
7	92.41 *±* 11.47	87.28 *±* 13.40	99.78 *±* 00.32	92.52 *±* 12.78	93.97 *±* 08.16	99.90 *±* 00.13
8	92.51 *±* 12.40	87.73 *±* 14.66	99.78 *±* 00.41	90.86 *±* 14.24	**96.22 ***±*** 06.61**	**99.94 ***±*** 00.12**
9	91.59 *±* 13.12	86.28 *±* 15.20	99.75 *±* 00.42	92.14 *±* 14.09	92.95 *±* 09.93	99.88 *±* 00.22
10	91.94 *±* 10.81	86.39 *±* 13.32	99.75 *±* 00.38	92.07 *±* 12.10	93.37 *±* 08.73	99.89 *±* 00.17
11	85.67 *±* 18.53	78.18 *±* 20.45	99.60 *±* 00.50	85.09 *±* 20.79	91.35 *±* 12.47	99.84 *±* 00.28
12	79.27 *±* 25.36	71.02 *±* 25.96	99.48 *±* 00.61	79.23 *±* 28.11	88.28 *±* 16.57	99.80 *±* 00.30
13	89.93 *±* 12.43	83.35 *±* 14.97	99.70 *±* 00.47	87.45 *±* 15.22	94.64 *±* 08.20	99.91 *±* 00.14
14	87.60 *±* 17.38	80.79 *±* 18.90	99.66 *±* 00.46	86.84 *±* 19.55	93.03 *±* 08.56	99.88 *±* 00.17
15	91.37 *±* 11.20	85.52 *±* 13.96	99.73 *±* 00.40	90.64 *±* 13.40	93.55 *±* 09.29	99.90 *±* 00.17
16	90.72 *±* 14.07	85.04 *±* 16.08	99.73 *±* 00.42	90.04 *±* 16.22	93.90 *±* 08.92	99.90 *±* 00.17
17	91.52 *±* 13.89	86.36 *±* 15.92	99.76 *±* 00.41	90.06 *±* 15.34	94.67 *±* 11.43	99.93 *±* 00.12
18	90.47 *±* 12.56	84.24 *±* 14.67	99.72 *±* 00.41	89.29 *±* 14.50	93.74 *±* 09.42	99.90 *±* 00.16
19	92.55 *±* 12.66	87.82 *±* 14.58	99.79 *±* 00.39	91.44 *±* 14.28	95.49 *±* 07.71	99.93 *±* 00.12
20	84.85 *±* 19.09	77.03 *±* 20.70	99.55 *±* 00.56	88.23 *±* 19.92	86.07 *±* 16.97	99.73 *±* 00.41
Minimum	79.27 *±* 10.20	71.02 *±* 12.25	99.48 *±* 00.30	79.23 *±* 11.43	86.07 *±* 06.61	99.73 *±* 00.12
Maximum	93.61 *±* 25.36	89.24 *±* 25.96	99.81 *±* 00.61	93.49 *±* 28.11	96.22 *±* 16.97	99.94 *±* 00.41
Mean	90.16 *±* 13.80	84.28 *±* 15.84	99.72 *±* 00.42	89.68 *±* 15.56	93.28 *±* 09.95	99.89 *±* 00.18

Performance analysis of the individual neural networks revealed substantial variability in predictive performance. Models 3 and 5 achieved the highest Dice scores of 93.61%. Conversely, Model 12 exhibited the lowest performance, with a Dice coefficient of 79.27%. The 14.34% difference between the best and worst results obtained using the same architecture highlights the instability of individual models and provides strong motivation for developing ensemble approaches. Furthermore, the high standard deviation of the Dice coefficient (13.80%) indicates the presence of clinically challenging cases in the dataset, for which the predictions of individual models become less reliable.

**Fig. 3:**
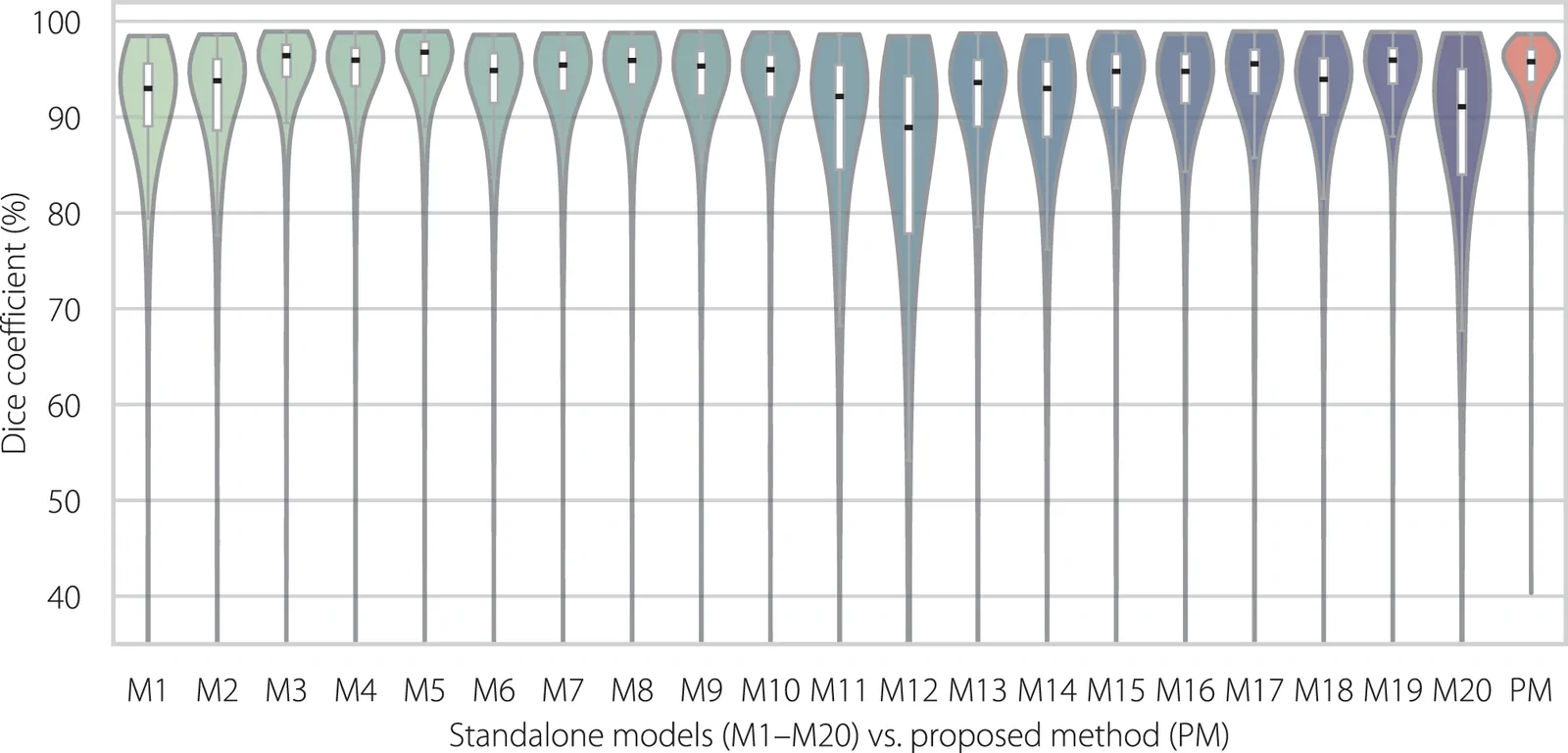
Performance variability analysis of individual baseline models vs the proposed method

A more detailed analysis of the performance variability among the standalone models across the test dataset is presented in Fig. 3 (columns M1–M20). While most models exhibit a characteristic top-heavy violin shape—indicating that the majority of test predictions cluster in the high-performance region above 90%—a distinct subset of models diverges from this pattern. Models 11 and 12 display markedly wider and more symmetric violin bodies, with substantial density extending below 70%, reflecting their large standard deviations of 18.53% and 25.36%, respectively. Model 20 similarly exhibits a heavier lower tail than the high-performing cluster. This visual separation suggests a bimodal structure within the initial model pool, comprising a dominant high-performance group and a distinct low-stability group, most plausibly attributable to convergence toward suboptimal local minima during stochastic gradient-based training.

The embedded box plots reveal a clear divergence in interquartile ranges. Stable models, such as Models 3, 4, and 5, exhibit narrow boxes located entirely above the 85% mark, with short whiskers. In contrast, the box plots for Models 11, 12, and 20 are substantially more elongated, with whiskers extending well below 60%. Their low-density tails extend into the 40%–55% range, corresponding to clinically challenging MRI slices with small or diffuse tumor regions. In these cases, the predictions of standalone models become highly unreliable.

The obtained results confirm that, although the Dilated U-net architecture is effective, its standalone use does not guarantee consistently high segmentation performance across all cases. Owing to the variability in individual model outputs, a hierarchical approach was proposed. The results presented in this section demonstrate how this approach mitigates errors associated with individual models.

In the second stage of the study, the effectiveness of classical ensemble methods was evaluated. Simple averaging and union-based aggregation methods were applied to the full set of 20 trained Dilated U-net models. The results are presented in Table  3. The comparative analysis shows that the choice of aggregation method significantly affects the final segmentation performance. Each approach has its own advantages and limitations in the context of medical image analysis.

**Table 3:** Ensemble performance for 20 models (*mean± SD*, %)

Method	Dice	Intersection over union	Accuracy	Recall	Precision	Specificity
Simple average	**93.33 ***±*** 09.19**	**88.47 ***±*** 11.57**	**99.80 ***±*** 0.32**	91.50 *±* 11.98	**96.60 ***±*** 03.61**	**99.95 ***±*** 0.06**
Union-based	85.94 *±* 15.57	77.71 *±* 17.69	99.48 *±* 0.60	**97.88 ***±*** 12.64**	78.06 *±* 17.86	99.48 *±* 0.60

The simple averaging method demonstrated a significant improvement in stability and accuracy compared to the average performance of the individual models. This method achieved a Dice coefficient of 93.33%, nearly identical to that of the best individual model (93.61%), while also exhibiting a substantial reduction in the standard deviation (9.19% vs 13.80%).

The union-based aggregation method, in turn, demonstrated high recall, reaching 97.88%. This approach identified virtually all pathological pixels detected by at least one model in the ensemble. However, this advantage was accompanied by reduced precision (78.06%) and a lower Dice coefficient (85.94%). The logical merging of masks led to oversegmentation, resulting in increased standard deviation and reduced overall segmentation performance across the remaining metrics.

Thus, the results presented in Table  3 demonstrate a trade-off between sensitivity and accuracy for the two methods. Simple averaging provides high boundary precision, whereas union-based aggregation ensures comprehensive pathology detection but introduces substantial noise. Therefore, the proposed hierarchical workflow aims to balance these approaches through multi-stage aggregation.

The final stage of the study was devoted to evaluating the proposed two-stage hierarchical workflow. The results for four different aggregation configurations (v1–v4) are presented in Table  4. The analysis confirms that the proposed two-stage ensemble method achieves higher segmentation performance than both the individual models and the two baseline ensemble methods. Variant 4, which combines simple averaging in the first stage with union-based aggregation in the second stage, demonstrated the best performance. This configuration achieved the highest Dice coefficient (94.50%) and IoU (89.91%), outperforming both the best individual models and the classical ensemble methods reported in Table  3.

**Table 4:** Performance comparison of four hierarchical ensemble configurations (*mean ± SD*, %)

Variant	Ensemble	Dice	Intersection over union	Accuracy	Recall	Precision	Specificity
1	Ensemble 1	94.26 *±* 08.48	89.95 *±* 10.27	99.83 *±* 00.23	93.45 *±* 09.95	96.10 *±* 05.25	99.94 *±* 00.09
	Ensemble 2	93.85 *±* 08.96	89.34 *±* 11.18	99.82 *±* 00.29	92.87 *±* 11.16	96.06 *±* 04.58	99.94 *±* 00.08
	Ensemble 3	89.42 *±* 15.80	83.28 *±* 17.30	99.72 *±* 00.42	87.42 *±* 18.11	95.17 *±* 05.33	99.92 *±* 00.11
	Ensemble 4	92.36 *±* 12.21	87.39 *±* 14.24	99.78 *±* 00.38	91.04 *±* 14.43	95.71 *±* 06.51	99.93 *±* 00.11
	Overall	92.36 *±* 12.26	87.39 *±* 14.20	99.79 *±* 00.36	90.07 *±* 14.68	**96.96 ***±*** 03.50**	**99.95 ***±*** 00.06**
2	Ensemble 1	92.39 *±* 09.84	86.89 *±* 11.70	99.76 *±* 00.27	98.06 *±* 08.16	87.96 *±* 11.93	99.78 *±* 00.26
	Ensemble 2	92.26 *±* 09.77	86.70 *±* 12.02	99.75 *±* 00.30	97.60 *±* 07.58	88.26 *±* 12.10	99.77 *±* 00.30
	Ensemble 3	88.60 *±* 13.11	81.29 *±* 15.36	99.60 *±* 00.49	94.66 *±* 12.97	84.65 *±* 15.20	99.67 *±* 00.42
	Ensemble 4	89.20 *±* 12.76	82.25 *±* 15.61	99.61 *±* 00.52	95.77 *±* 11.63	85.16 *±* 14.89	99.68 *±* 00.45
	Overall	86.17 *±* 15.05	77.90 *±* 17.27	99.48 *±* 00.59	**98.04 ***±*** 12.00**	78.28 *±* 17.44	99.48 *±* 00.60
3	Ensemble 1	92.39 *±* 09.84	86.89 *±* 11.70	99.76 *±* 00.27	98.06 *±* 08.16	87.96 *±* 11.93	99.78 *±* 00.26
	Ensemble 2	92.26 *±* 09.77	86.70 *±* 12.02	99.75 *±* 00.30	97.60 *±* 07.58	88.26 *±* 12.10	99.77 *±* 00.30
	Ensemble 3	88.60 *±* 13.11	81.29 *±* 15.36	99.60 *±* 00.49	94.66 *±* 12.97	84.65 *±* 15.20	99.67 *±* 00.42
	Ensemble 4	89.20 *±* 12.76	82.25 *±* 15.61	99.61 *±* 00.52	95.77 *±* 11.63	85.16 *±* 14.89	99.60 *±* 00.45
	Overall	92.16 *±* 10.84	86.71 *±* 12.72	99.75 *±* 00.32	95.83 *±* 11.24	90.28 *±* 09.47	99.82 *±* 00.24
4	Ensemble 1	94.26 *±* 08.48	89.95 *±* 10.27	99.83 *±* 00.23	93.45 *±* 09.95	96.10 *±* 05.25	99.94 *±* 00.09
	Ensemble 2	93.85 *±* 08.96	89.34 *±* 11.18	99.82 *±* 00.29	92.87 *±* 11.16	96.06 *±* 04.58	99.94 *±* 00.08
	Ensemble 3	89.42 *±* 15.80	83.28 *±* 17.30	99.72 *±* 00.42	87.42 *±* 18.11	95.17 *±* 05.33	99.92 *±* 00.11
	Ensemble 4	92.36 *±* 12.21	87.39 *±* 14.24	99.78 *±* 00.38	91.05 *±* 14.43	95.71 *±* 06.51	99.93 *±* 00.11
	Overall	**94.50 ***±*** 04.89**	**89.91 ***±*** 07.42**	**99.83 ***±*** 00.18**	96.59 *±* 04.14	92.96 *±* 07.26	99.87 *±* 00.16

The main advantage of Variant 4 is not only its improved accuracy but also a significant increase in result stability. In this configuration, the standard deviation of the Dice coefficient decreased to a record low of 4.89%. This is 5.31 percentage points lower than the minimum value observed for the individual models in Table 2 and 4.30 percentage points lower than that of the simple averaging method reported in Table 3. These findings confirm the high stability of the proposed hierarchical approach. Owing to these advantages, the method produces reliable results even in complex clinical cases where individual neural networks may exhibit substantial errors.

A comparative analysis of the other variants of the proposed method also provides important insights into segmentation optimization. Variant 1, which uses simple averaging at both stages, demonstrated high overall accuracy and specificity but was less effective at detecting small tumor regions than Variant 4. Variants 2 and 3, which use union-based aggregation at different stages, achieved recall values of up to 98.04%. However, these configurations were associated with oversegmentation and lower final Dice coefficients. Therefore, optimal performance is achieved by using simple averaging in the first stage to suppress individual model noise and union-based aggregation in the second stage for final mask fusion.

Thus, Variant 4 demonstrates superior performance to the traditional methods. The achieved accuracy and prediction stability suggest that this workflow is a promising approach for automated MRI analysis in clinical practice.

The rightmost column of Fig. 3 illustrates the core contribution of the proposed method. This profile demonstrates how the framework overcomes the previously identified instabilities of the standalone models. The violin plot for the proposed method is visibly more compact than those of the individual models. Its density is concentrated tightly above 88%, resulting in a substantially reduced lower tail.

Furthermore, the embedded interquartile box is positioned higher and is more compact than those of even the top-performing individual models (Models 3 and 5). This visual evidence directly corroborates the quantitative results. Specifically, the proposed method achieves a record-low standard deviation of 4.89%, compared with 10.20%–25.36% for the individual baseline models. Overall, these distribution profiles suggest that the hierarchical aggregation configuration effectively mitigates localized failure modes. The framework substantially reduces individual model errors rather than merely diluting them through linear averaging.

The results of the ablation study evaluating grouping strategies and ensemble orders are presented in Table  5. The data demonstrate that extreme configurations lead to lower overall segmentation performance. Combining all 20 models into a single group yielded the lowest performance (mean Dice coefficient: 93.33%). Conversely, creating numerous small groups (e.g., 10 groups of 2) also resulted in suboptimal performance (mean Dice coefficient: 93.76%).

**Table 5:** Ablation study of grouping strategies and ensemble chronological orders

Configuration	Mean Dice (%)	Max Dice (%)	SD (Order variation, %)
1 group of 20	93.33	93.33	*±* 0.00
2 groups of 10	93.78	93.98	*±* 0.14
4 groups of 5 (proposed)	**94.18**	**94.29**	*±* **0.11**
5 groups of 4	93.88	94.02	*±* 0.11
10 groups of 2	93.76	93.99	*±* 0.26

The proposed configuration of 4 groups of 5 models achieved the highest performance across random permutations of the chronological model order (mean Dice coefficient: 94.18%). Furthermore, the resulting standard deviation of 0.11% indicates that the proposed hierarchical aggregation is robust and invariant to model ordering. It should be noted that specific chronological groupings, such as the sequential order used in the primary evaluation, yielded a peak Dice coefficient of 94.50%. These results indicate that, although the ensemble mechanism is structurally stable with respect to sequence variations, the selected *4 × 5* configuration maximizes the upper bound of segmentation accuracy.

**Fig. 4:**
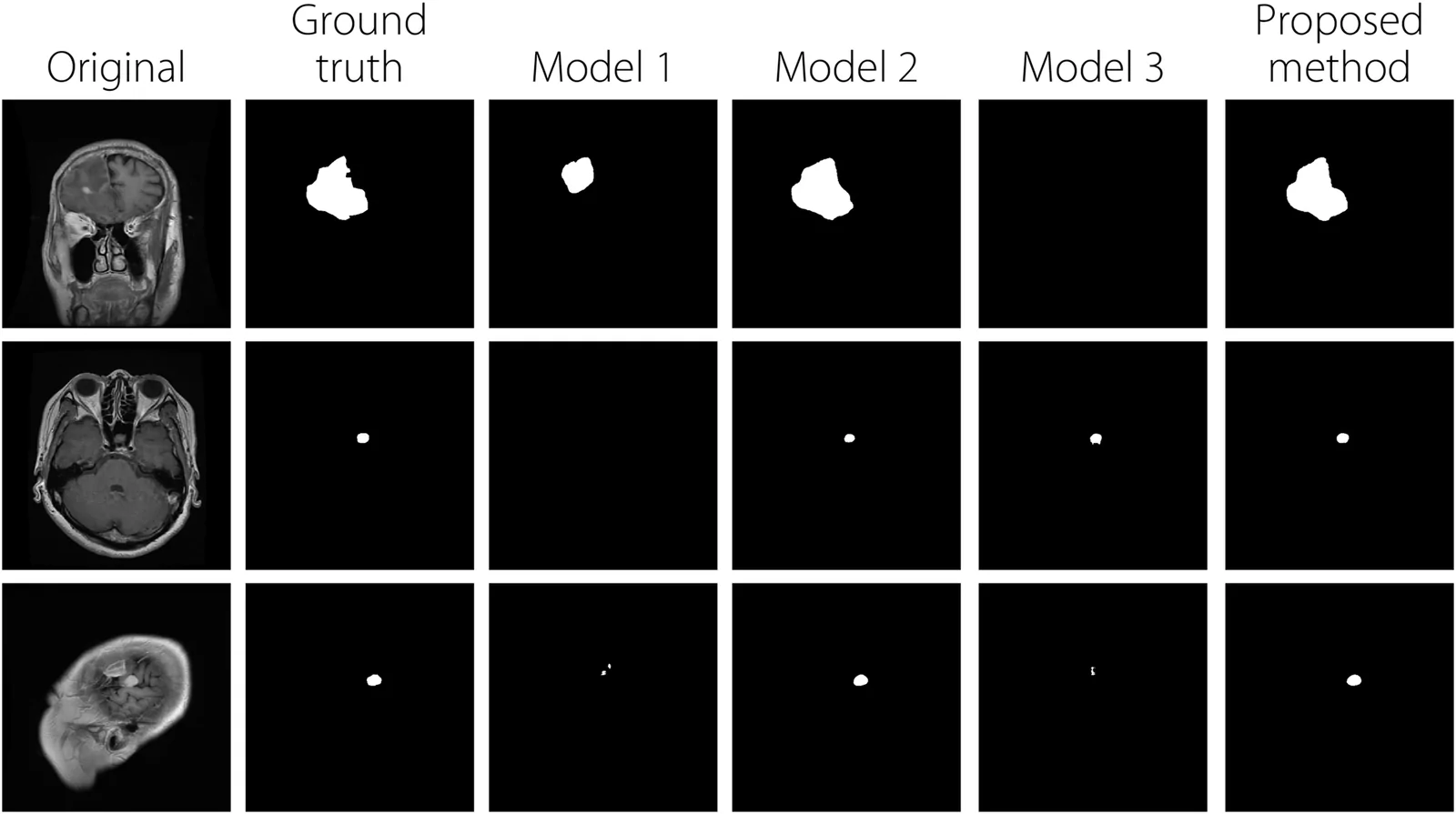
Comparison of segmentation results across different methods

A visual comparison of the segmentation results obtained using different approaches is presented in Fig. 4. The figure includes the ground truth, the predictions of three representative individual models, and the final result produced by the proposed ensemble method. To provide a representative evaluation spanning the performance variability of the 20 models, Models 1, 2, and 3 were selected based on their statistical characteristics: Model 1 represents the moderate-performance tier, Model 2 represents the mean individual performance, and Model 3 represents the top-performing tier. Extreme outliers, such as Model 12, were excluded from the visualization to focus on typical boundary ambiguities. As shown in the figure, these individual models produce segmentation masks with noticeable inaccuracies, including missing tumor regions and false positives. In contrast, the proposed method successfully overcomes these limitations, providing more precise tumor localization and smoother boundaries.

The baseline Dilated U-net architecture contains approximately 31.05 million parameters per model. The complete 20-model ensemble requires approximately 2.32 GB of disk storage (621.1 million cumulative parameters, corresponding to 118.62 MB per individual .h5 weights file). However, the sequential nature of the inference pipeline ensures that only one model needs to be loaded into GPU memory at any given time. Consequently, the active VRAM footprint remains strictly bounded while maintaining high segmentation accuracy.

Each of the 20 constituent models was trained using the same set of 2451 training images with different random seeds to ensure prediction diversity. On the local NVIDIA GeForce RTX 4060 Ti GPU, training a single base model required an average of 154.05 minute and typically terminated early at approximately the 27th epoch. Consequently, the total training time for the entire ensemble was approximately 51.35 hour. Because the base models are completely independent and have no interdependencies, the training process can be readily distributed across multiple GPUs, substantially reducing the overall training time.

To assess the feasibility of practical clinical application in computer-aided diagnostic (CAD) systems, the segmentation time of the proposed architecture was evaluated. A key performance requirement for CAD systems is the computational time required to process a single patient slice. Processing a single T1-CE MRI slice sequentially through all 20 models requires 1122.80 milliseconds (56.14 ms per individual model). The multi-stage ensemble aggregation adds an overhead of 111.44 milliseconds, resulting in a total inference time of 1234.24 milliseconds per slice. This sequential execution pipeline, combined with explicit memory flushing (K.clear_session()), was strictly enforced to prevent VRAM allocation conflicts within the memory limitations of a single consumer GPU. During inference, the peak dynamic VRAM allocation reached 4925.89 MB (approximately 4.81 GB) because of the temporary storage of intermediate feature maps. In a production clinical environment, deployment solutions such as NVIDIA Triton Inference Server can further reduce inference latency through dynamic batching, concurrent execution, and multi-GPU processing.

To demonstrate the scientific significance and competitiveness of the proposed method, the obtained results were compared with state-of-the-art segmentation methods. Table  6 presents studies that used the same Figshare dataset, ensuring a fair and meaningful comparison. Key performance metrics, including the Dice coefficient, IoU, and other measures, were used for the evaluation.

**Table 6:** Performance comparison of the proposed method against state-of-the-art approaches (%)

Reference	Dataset	Dice	Intersection over union	Accuracy	Recall	Precision	Specificity
Sobhaninia et al. [26]	Figshare	80.03	-	-	-	-	-
Díaz-Pernas et al. [27]	Figshare	82.80	-	-	-	94.00	-
Mayala et al. [18]	Figshare	84.69	74.43	-	81.91	-	99.85
Razzaghi et al. [45]	Figshare	86.02	-	-	-	-	-
Gupta et al. [43]	Figshare	-	86.65	-	-	-	-
Wang et al. [44]	Figshare	92.88	-	-	90.20	91.50	-
Allah et al. [42]	Figshare	89.28$^{1}$	-	-	-	-	-
Sahoo et al. [46]	Figshare	90.20	-	99.60	90.20	90.50	99.80
Preetha et al. [23]	Figshare	93.39	87.95	99.79	91.00	**96.57**	99.63
Proposed method	Figshare	**94.50**	**89.91**	**99.83**	**96.59**	92.96	**99.87**

^1^The Edge U-Net model was originally evaluated on a multiclass segmentation task. We report its average Dice coefficient to provide broader methodological context

According to the data presented in Table  6, the proposed method outperforms all considered approaches across most metrics. The Dice coefficient of 94.50% is the highest among the reported studies, surpassing the method of Preetha et al. [23] (93.39%) and substantially exceeding the earlier work of Sobhaninia et al. [26] (80.03%). Furthermore, the proposed method demonstrates excellent performance compared with advanced architectures designed to address boundary ambiguity. Allah et al. [42] proposed the Edge U-net framework, which uses contrast limited adaptive histogram equalisation, a specialized edge guidance block, and a custom boundary-enhanced loss function to improve tumor localization. Their approach achieved an average Dice coefficient of 89.28% across three tumor classes. Our hierarchical ensemble resolves boundary uncertainties through its inter-group union strategy without requiring heavily customized loss functions or additional edge-extraction modules. The proposed framework also outperforms other sophisticated methods, including the recurrent residual U-net proposed by Gupta et al. [43] (IoU of 86.65%) and the Transformer-based MSegNet proposed by Wang et al. [44] (Dice coefficient of 92.88%).

The advantage of the proposed method over the considered approaches is primarily attributed to the use of dilated convolutions in the Dilated U-net architecture. Notably, even a single individual model (Model 5) achieved a Dice coefficient of 93.61%, which exceeds the result reported by Preetha et al. [23] (93.39%).

An additional factor contributing to the performance improvement is the ensemble approach. When developing segmentation methods, it is important to consider not only the absolute values of the evaluation metrics but also their variability, which can be assessed using the standard deviation. A lower standard deviation indicates more consistent model performance across different medical images. In this case, the standard deviation of 4.89% indicates that the proposed method exhibits relatively stable performance.

To validate the effectiveness and empirical necessity of the post-processing stage, an ablation analysis was conducted on the test images. The performance of the proposed hierarchical ensemble was evaluated with and without the post-processing stage. Integrating this step successfully suppressed false-positive noise outside the main tumor mass, as quantitatively confirmed by an increase in the mean precision from 92.81% to 92.96%. Consequently, the overall segmentation quality improved, increasing the mean Dice coefficient from 94.43% *±* 5.03% to 94.50% *±* 4.89% and the IoU from 89.81% *±* 7.61% to 89.91% *±* 7.42%. The primary value of this stage lies in anatomical regularization and improved worst-case stability. Crucially, the reduction in the standard deviation across all major metrics demonstrates that the post-processing pipeline successfully reduces variability and enhances overall prediction stability.

To facilitate clinical integration, the model weights can be converted to the open neural network exchange format. This enables cross-platform compatibility and efficient inference on standard hospital hardware. Deployment can be simplified by containerizing the pipeline using Docker, thereby eliminating environment dependency issues. In practical workflows, data transmission would use standard DICOM routing protocols to communicate directly with an existing picture archiving and communication system. This configuration allows the framework to function as a standard medical imaging node without disrupting routine hospital operations.

### Visual inspection of misclassified cases

**Fig. 5:**
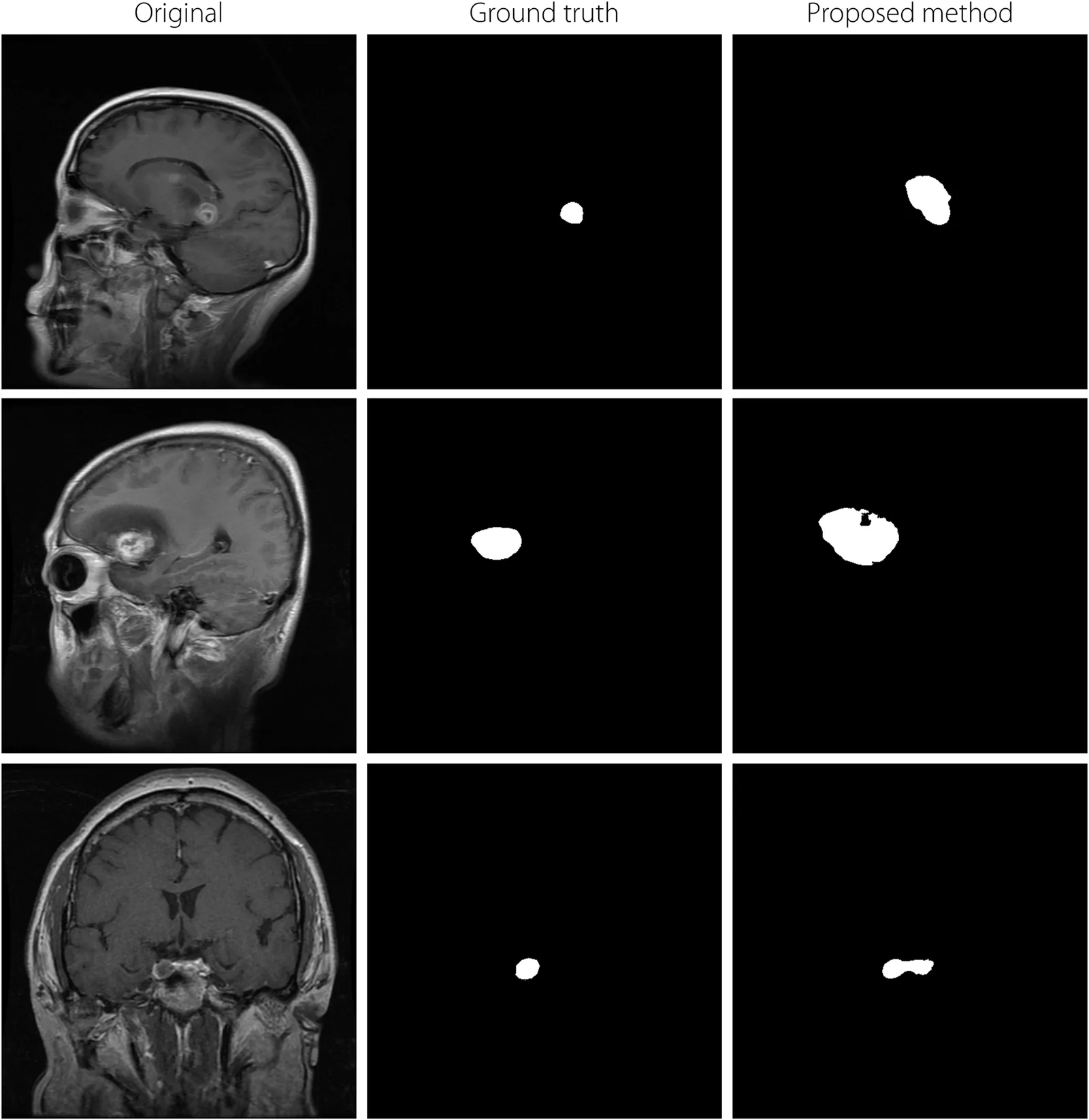
Analysis of worst-case segmentation results

To analyse the limitations of the proposed method, underperforming test cases with a Dice coefficient below 85% were selected for qualitative evaluation. The comparative visualization in Fig. 5 presents the original T1-CE MRI scans, the corresponding ground truth, and the model predictions. Based on visual inspection of segmentation overlays and intensity characteristics, these cases were classified into several error categories.

The first category is boundary under-segmentation. This type of error results in a small reduction in the predicted tumor volume, with an average difference of 9.62% relative to the ground truth. These discrepancies are mainly observed in peripheral tumor regions, where the intensity contrast between the lesion and surrounding tissue is reduced, making boundary delineation more difficult.

The second category is boundary over-segmentation. In these cases, the predicted tumor region is larger than the ground truth, with an average volume increase of 55.04%. Such errors are typically observed in regions adjacent to hyperintense anatomical structures, including vessels and dural regions, which may exhibit similar intensity patterns on contrast-enhanced images.

Although these boundary-related errors are present in a subset of cases, the proposed framework reduces the occurrence of severe segmentation failures. For example, complete omission of tumor regions was not observed in the evaluated test set. Overall, the hierarchical aggregation approach reduces the variability of predictions from individual models and improves the stability of the final segmentation output.

### Statistical analysis

A one-way analysis of variance was performed to evaluate segmentation performance across the proposed method, the simple averaging ensemble, and the best-performing single model (Model 5). The analysis revealed a statistically significant difference among the evaluated approaches (F(2, 1836) *=* 3.138, *p* *=* 0.0436), confirming that the choice of aggregation strategy significantly affects the Dice coefficient.

To further investigate the source of this significance, a post-hoc pairwise analysis was conducted. Specifically, a paired *t*-test was employed to compare the proposed method directly with the best-performing individual model (Model 5). The resulting *p*-value of 0.0259 (*p < 0.05*) indicates that the performance improvement achieved by the hierarchical architecture is statistically significant.

## Conclusions

This study developed and evaluated a two-stage hierarchical ensemble method based on deep neural networks for semantic segmentation of brain tumors in MRI scans. The use of the Dilated U-net architecture as a core component enabled effective incorporation of the spatial context of pathological regions through dilated convolutions, which is critically important for accurate delineation of tumor boundaries. Experimental evaluation of 20 independent models confirmed the strong baseline performance of the selected architecture; however, the observed significant variability in individual model results justified the need for more advanced aggregation strategies to improve stability and robustness.

The key finding of this study is the demonstrated superiority of the proposed hierarchical approach (Variant 4) over classical ensemble methods, such as simple averaging and union-based aggregation. This configuration corresponds to the proposed method and involves intra-group averaging of binary masks followed by a final logical union. Applying this method to the Figshare dataset resulted in a Dice coefficient of 94.50%. Of particular note is that the proposed method reduced the standard deviation of the metrics by approximately half compared with single models, indicating high stability with respect to input variability.

Comparative analysis with state-of-the-art methods showed that the proposed method achieved higher values for key metrics than the considered approaches. The use of post-processing at each level of the hierarchy reduced the number of misclassifications and improved the stability of the results.

### Limitations

A primary limitation of the current study is that the proposed hierarchical ensemble is specifically formulated for binary segmentation tasks on a single, albeit comprehensive, dataset. Although the dataset comprises diverse pathological subtypes (glioma, meningioma, pituitary adenoma), subtype-specific performance metrics were not independently evaluated. Furthermore, the framework has not yet been validated across diverse multi-center datasets, which remains a critical requirement for broader clinical deployment.

Methodologically, the design relies on a strategic combination of established methods—specifically, Dilated U-net, simple within-group averaging, and between-group logical union. Consequently, the main aggregation strategy relies entirely on static, predefined rules. While this approach successfully minimizes stochastic errors and preserves boundary completeness without adding structural complexity, it does not allow dynamic adjustment of individual model weights based on localized image features.

Finally, although the current framework satisfies baseline hardware constraints, the sequential execution of the ensemble limits its real-time segmentation capabilities.

### Future work

To enhance the clinical applicability and methodological depth of the proposed approach, future research will focus on several key directions.

First, the framework will be validated on larger-scale, multi-center datasets, and the architecture will be extended to multiclass segmentation to assess its robustness across distinct histopathological subtypes.

Second, to address current methodological limitations and accelerate real-time inference, future studies will explore advanced dynamic aggregation mechanisms, including adaptive weighting, attention-guided ensemble fusion, and heterogeneous ensembles comprising diverse lightweight architectures.

Additionally, future work will investigate advanced preprocessing pipelines, generative models (e.g., generative adversarial networks and diffusion models) for data augmentation, and multi-modal fusion (combining complementary MRI modalities such as T1, T2, and FLAIR) to further improve model generalization.

## Data Availability

The datasets generated and/or analysed during the current study are available in the GitHub repository: https://github.com/riggelllll/Brain-Tumor-Segmentation-Ensemble.

## Abbreviations

CAD: Computer-aided diagnostic
IoU: Intersection over union
MRI: Magnetic resonance imaging
TP: True positives
TN: True negatives
FP: False positives
FN: False negatives
